# Nutritional determinants, childhood morbidity patterns, and healthcare utilization among Chinese children: a population-based study

**DOI:** 10.3389/fpubh.2026.1909080

**Published:** 2026-09-08

**Authors:** Pengli Wang, Wenhua Liao, Li Zhang, Mingjia Zhao, Dong Zhang, Nanxiang Huang

**Affiliations:** Department of Pediatric Surgery, Beijing Anzhen Nanchong Hospital, Capital Medical University and Nanchong Central Hospital, The Second Clinical Medical School of North Sichuan Medical University, Nanchong, Sichuan, China

**Keywords:** childhood nutrition, Chinese children, dietary quality, healthcare utilization, malnutrition, morbidity burden

## Abstract

**Background:**

Childhood nutrition has a substantial impact on growth, disease susceptibility, and use of health care services. Malnutrition is a double burden in China, characterized by chronic undernutrition and childhood obesity.

**Objective:**

The objective of this study was to explore the relationships between nutrition determinants, childhood morbidity patterns and health care utilization in the Chinese population.

**Methodology:**

This retrospective, institution-based study analyzed routinely collected health records from 720 children aged 3–14 years who received care at a tertiary healthcare center in Nanchong, Sichuan, China, between 2021 and 2025. Nutritional status and dietary quality were determined by using the routine health records. Morbidity burden and service use were assessed using medical records for children. Independent predictors were identified by using multivariable logistic regression.

**Results:**

15.1% of participants were undernourished, and 20.3% were overweight or obese. The childhood morbidity burden was observed in 25.7% of children and was significantly higher in children who were undernourished and obese (*p* < 0.001). Known-groups validity testing showed that all 9 individual components of the dietary-quality score varied significantly and monotonically across derived tiers (Cochran-Armitage *p* < 0.001 for all), supporting the internal coherence of the measure. Poor dietary quality, as measured by this study-specific composite index, was an independent risk factor for a high burden of morbidity (Adjusted odds ratio (AOR) = 2.76, 95% CI: 1.89–4.05). High healthcare utilization was observed in 25.8% of participants. It was independently associated with high morbidity burden (AOR = 3.87, 95% CI: 2.71–5.54), undernutrition (AOR = 2.09, 95% CI: 1.37–3.20), obesity (AOR = 2.34, 95% CI: 1.40–3.89), poor dietary quality (AOR = 1.96, 95% CI: 1.32–2.92), low household income (AOR = 1.58, 95% CI: 1.07–2.34), and grandparent caregiving (AOR = 1.45, 95% CI: 1.01–2.11).

**Conclusion:**

Undernutrition and overweight/obesity, in addition to poor diet quality, are associated with higher morbidity rates and utilization of health services in children in China. The findings suggest that nutritional status and dietary quality are significantly associated with childhood morbidity burden and healthcare utilization.

## 1 Introduction

Health status in childhood is one of the major determinants of population health and human capital formation. China has seen significant shifts in child nutrition as a result of rapid economic growth, urbanization and nutrition transition. Although there has been significant progress in reducing extreme undernutrition, the country still faces several ongoing nutrition issues. Undernutrition, such as stunting and micronutrient deficiencies, persists in some rural and low-income groups, and overweight and obesity have risen significantly in children in urban areas (1). The simultaneous presence of both under- and over-nutrition has created the dual burden of malnutrition in Chinese children. Childhood nutritional status has been linked to physical growth, cognitive development, immune function, and health outcomes throughout childhood (2). Evidence suggests that a good diet, good nutritional status, and food security are associated with better child health outcomes. In contrast, a poor diet, poor food security, and nutritional deficiencies are associated with adverse child health outcomes. Nutritional determinants can have short- and long-term health impacts, as childhood is a time of growth and development. As a result, nutrition and child health are significant public health issues in China (3).

Nutritional determinants are well linked to patterns of childhood morbidity through biological, environmental, and social channels. Good nutrition is known to play a role in resisting disease and in ensuring healthy growth and development. In contrast, inadequate nutrition has been linked to an increased risk of infectious and non-communicable diseases. A high burden of respiratory infections, gastrointestinal infections, anemia, poor growth, and recurrent disease episodes is more common in children with inadequate dietary intake, micronutrient deficiencies, or malnutrition. Concurrently, overconsumption of energy-dense foods has been correlated with childhood overweight and obesity, and they are related to metabolic disorders and other chronic health conditions (4). Significant correlations have been established between nutrition status and commonly occurring child diseases such as respiratory tract infection, diarrheal disease, allergic disease and hospitalization risk. Additionally, nutritional status and disease rates are correlated with household income, parents’ education, access to health services, and urban–rural living. Dysfunctional households might have less access to medical services and health care and lack the resources to purchase good food, making the child more susceptible to sickness. These results highlight the importance of nutritional factors in shaping children’s morbidity patterns and their potential role in explaining health disparities between groups (5).

Several mechanisms may account for the relationships between nutritional determinants, childhood morbidity, and healthcare utilization. Adequate intake of essential macronutrients and micronutrients is correlated with proper immune system development, disease resistance, and quick recovery from illness. Protein, iron, zinc, vitamin A and vitamin D are necessary to maintain immune competence and growth. These nutrients have been linked to decreased immune function, higher risk of infection, longer illness, and greater disease severity when deficient (6). On the other hand, high caloric consumption, obesity, and associated health complications have been linked to chronic inflammation and metabolic disorders. In these pathways, nutritional status can affect the incidence, intensity and duration of disease events in children (7). Children may have to come to the clinic more often, be admitted to the hospital, use more drugs and other health care services as their morbidity rises. If the nutritional status is compromised, it is likely to extend the recovery period further and increase healthcare costs. Hence, health care utilization can be considered as an important outcome related to both nutritional determinants and the burden of childhood diseases (8).

Knowledge of the link between childhood nutrition and health has grown, but gaps remain. Most previous research has studied nutritional status, dietary practices, childhood morbidity, or health service use as individual outcomes. It has generally been conducted within specific disease groups, age groups, or geographic populations. Therefore, little is known about the joint association between nutritional status and dietary quality with the overall morbidity burden and patterns of healthcare utilization in Chinese children. Furthermore, the independent associations of nutritive and socioeconomic factors with these inter-related outcomes have not been fully examined in the same pediatric population. Based on the existing evidence, the present study aims to add nutritional status, dietary quality, morbidity burden, healthcare utilization, and household characteristics to a population-based analytical framework using routinely collected pediatric health information from a tertiary healthcare centre in Sichuan, China. The results from the study might yield potentially relevant information about the situation of the children receiving care in this setting. However, the study sample was not intended to be nationally representative of children in China more generally. Thus, the purpose of this study was to assess the correlation between nutrition determinants, childhood morbidity patterns, and utilization of health services among children in China, and to explore the association between high morbidity burden and high health service utilization that is independent of nutrition determinants and childhood morbidity patterns.

## 2 Methodology

### 2.1 Study design and setting

This is a single-centre, retrospective, record-based study that investigated associations between nutrition determinants, childhood morbidity patterns, and health care utilization among children treated at Beijing Anzhen Nanchong Hospital, Sichuan, China. Children’s health records were extracted from the database using routinely collected data from 1 January 2021 to 31 December 2025. It is a tertiary hospital for children in the cities and neighboring rural areas of Nanchong, Sichuan Province.

The study population comprised children whose data were available in the institutional databases and was not drawn from a probability-based national or province-wide sampling. Thus, although the sample comprised children from both urban and rural residential backgrounds, it is not representative of all children in China. The results are largely based on the associations identified among children receiving care at the health care site. The study was reported following the Strengthening the Reporting of Observational Studies in Epidemiology (STROBE) Statement (9).

### 2.2 Study population

The target population included children aged 3–14 years who had been routinely assessed for child health status and had data on nutrition, morbidity, and health care utilization in institutional databases during the study period. The source population comprised children who attended or received documented healthcare services at the participating tertiary healthcare center during the study period. Children who did not seek care at the institution, received care exclusively elsewhere, or lacked accessible institutional records were not represented in the study cohort. To be included, children had to have complete records of anthropometric measurements, demographic data, household data, morbidity history, and indicators of healthcare utilization. Children were excluded who had severe congenital anomalies, genetic syndromes that impact growth, terminal illness, an incomplete nutritional assessment, duplicate records, or missing outcome information.

All eligible children with complete information on the primary exposure variables, morbidity outcomes, and healthcare-utilization outcomes during the study period were included. No additional sampling or participant selection was performed after application of the predefined eligibility criteria. The study used a consecutive census of all eligible records available from the participating institutions during the predefined study period rather than probability-based sampling.

### 2.3 Sample selection and participant flow

**Figure 1:**
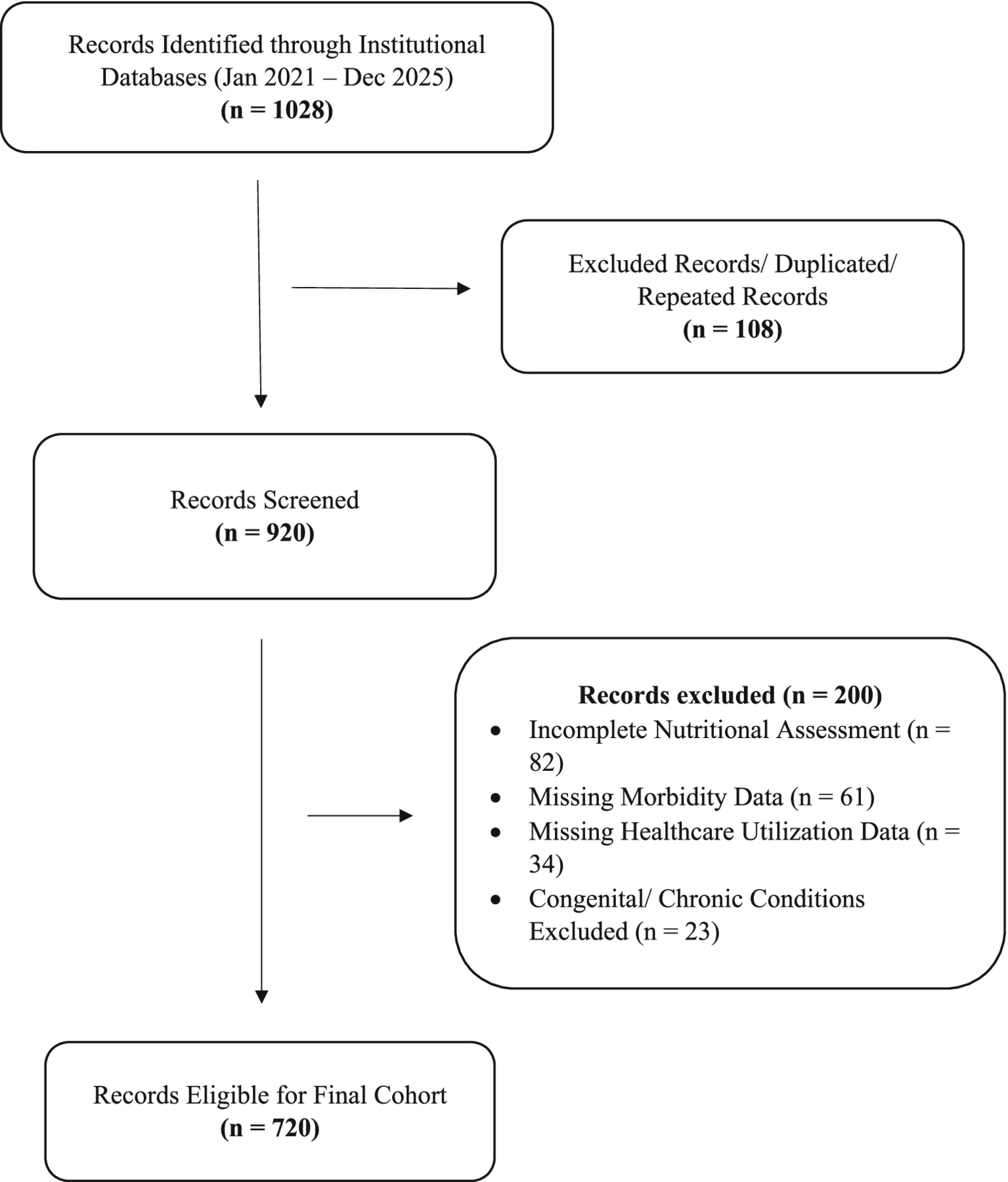
Participation flow diagram.

To recruit eligible participants, a multistage retrospective screening process was conducted. Electronic databases from participating tertiary healthcare center initially identified 1,028 pediatric records of children aged 3–14 years who attended routine health assessments or received healthcare services between January 2021 and December 2025. After removal of 108 duplicate records and repeat visits, 920 unique participants remained. Subsequently, 82 records were excluded because of incomplete anthropometric or nutritional information, 61 due to insufficient healthcare utilization documentation, and 34 because of missing morbidity records. An additional 23 children with congenital disorders or chronic conditions known to affect nutritional status and growth trajectories substantially were excluded. Following application of all eligibility criteria, the final analytical cohort comprised 720 children and was included in the statistical analyses (Figure 1). The final analytical sample therefore represented all eligible records available within the institutional databases during the study period rather than a randomly selected or population-representative sample.

None of the calculations of the *a priori* required sample size was performed since the study was retrospective, based on all eligible records available from the participating institutional databases within the specified study period. Thus, the final sample size was based on the number of records eligible for the study, not on participants who would be recruited. After excluding children who did not meet the criteria, however, the final analytical cohort consisted of 720 children, 185 of whom had high morbidity burden and 186 had high healthcare utilization. The number of outcome events was deemed to be sufficient to perform the multivariable logistic regression analyses and included the selected predictor variables without compromising the events-per-variable ratio.

The study population comprised all eligible children with complete records available at the participating tertiary healthcare centre during the study period and was not derived through national or probability-based sampling.

### 2.4 Data collection procedures

Experienced researchers collected the data using a standardized data abstraction sheet. Data from the participating tertiary healthcare center were collected from child health surveillance, nutritional assessment, outpatient, immunization and healthcare-utilization databases. Children living in both urban and rural areas were included in the institutional databases. However, the geographic distribution of the participants did not represent a sampling frame across the province or the country, but rather the hospital’s service catchment area. Two independent investigators abstracted data from the eligible institutional health records using a standardized data-abstraction form to ensure consistency and reliability. Any discrepancies were resolved through discussion and consensus with a senior investigator. A comprehensive data quality control process was performed prior to statistical analysis to identify implausible values, missing data, and inconsistencies.

During participant-selection process, records with missing information for the primary outcomes (morbidity burden or health care utilization) were not included. Records that did not include any of the anthropometric or nutritional details needed for the classification of nutritional status and dietary quality were also omitted. The analytical data set was complete for the main study outcomes and variables in the multivariable regression models after applying the eligibility criteria. Hence, no multiple imputation or other statistical imputation method was applied. Complete case analysis was performed. All extracted records were de-identified before analysis, and access to the study dataset was restricted to authorized members of the research team to protect participant confidentiality.

Selection bias due to the inclusion of children for whom health records were available and complete, and information bias due to the use of routinely collected clinical data, and potential misclassification of dietary behaviors and/or morbidity events were possible sources of bias. All these risks were reduced by standardizing data-abstraction processes, having two investigators cross-check all the data independently, and reaching consensus regarding any discrepancies. However, such measures could not account for all potential sources of bias in retrospective record-based studies.

### 2.5 Nutritional assessment

Nutritional status was assessed using standardized anthropometric parameters routinely collected during child health examinations. Body weight, height and body mass index (BMI) were measured. Nutritional classification was conducted using the age- and sex-specific growth reference recommended for Chinese children (10). The categories of nutritional status were undernutrition, normal nutritional status, overweight and obesity. Where appropriate, anthropometric indices were converted to standardized z-scores to allow for comparisons between age groups.

A study-specific composite score was used to assess the quality of the child’s diet and included 9 dietary components commonly recorded in the institutional child health record. The components consisted of daily fruit intake, daily vegetable intake, dairy consumption, regular breakfast consumption, whole grain consumption, adequate dietary diversity, sugar-sweetened beverage consumption, fast food consumption, processed snack consumption, and age-appropriate healthy dietary practices. The components were evaluated on a score of 0 or 1 on set criteria. A favorable dietary behavior received 1 point if it was met and 0 if it was not met. Dietary behaviors that were potentially adverse were reverse scored, with zero points given for higher consumption and one point for lower consumption of the behavior; for example, higher consumption of sugar-sweetened beverages was assigned zero points and lower consumption was assigned one point.

Component scores were added together to provide an overall score of dietary quality (0 to 9, with higher scores reflecting healthier dietary patterns). Participants were grouped into poor dietary quality (0 to 2 points), moderate dietary quality (3 to 5 points) and good dietary quality (6 to 9 points). These food pattern categories were established *a priori* to identify relatively poor, moderate, and good dietary patterns in the study participants. The thresholds and scoring for each component are detailed in Supplementary Table S1.

The dietary components were chosen to represent dietary practices relevant to Chinese children. They were conceptually based on the Chinese Children Dietary Index, which was designed based on the Chinese Dietary Guidelines and validated for measuring overall diet quality in Chinese school-aged children (11). However, the original Chinese Children Dietary Index could not be directly used because some dietary indicators required for the index were not routinely documented in the institutional health records used in this study. Therefore, a study-specific 9-component composite dietary quality score was generated by combining the available dietary indicators. This score should be considered an indicator of dietary quality in this specific context and not the validated Chinese Children Dietary Index. To further evaluate the coherence of the composite score, known-groups validity was assessed by testing whether the prevalence of each individual dietary component varied significantly and monotonically across the derived Poor, Moderate, and Good tiers.

### 2.6 Assessment of childhood morbidity patterns

Physician-diagnosed childhood diseases documented in the included health care databases were used to measure childhood morbidity. Morbidity categories were respiratory infections, gastrointestinal disorders, allergic diseases, nutritional deficiency disorders, dermatological conditions, ear, nose and throat conditions, asthma or wheezing disorders, oral and dental conditions, recurrent febrile illnesses and other documented acute illnesses. All physicians diagnosed illness episodes were counted only once. If the same illness episode was documented during follow-up, it was not considered as an additional instance of the same illness.

The total number of physician-diagnosed morbidity episodes was then divided by the observation period for each participant and reported as episodes per year. The morbidity burden was classified as low (0–1 episode/year), moderate (2–4 episodes/year) or high (≥5 episodes/year). These categories were *a priori* defined for analytical purposes to differentiate infrequent, intermediate and recurrent morbidity among children. The thresholds were based on the distribution and clinical interpretation of documented morbidity frequency and not on validated clinical severity categories. High morbidity burden (defined as ≥5 episodes/year) was evaluated as a binary variable, and low and moderate morbidity were considered the control group.

### 2.7 Assessment of healthcare utilization

Data on healthcare utilization included outpatient consultations, emergency department (ED) visits, hospital admissions, preventive health visits, vaccination-related visits, specialist consultations, telehealth consultations, and electronic health records. One health care visit per incident was recorded. If there were several healthcare encounters within the same illness episode, an encounter was included if it was a different use of healthcare services.

The number of healthcare encounters each participant had been totaled and annualized based on the length of available follow-up. Healthcare-utilization rate was annualized (total number of documented healthcare encounters/individual observation period, in years). Participants were then divided into three groups of healthcare utilization: low, 0–7 encounters; moderate, 8–13 encounters; and high, ≥14 encounters. These thresholds were established prior to multivariable analyses and were chosen to differentiate between groups of children with relatively little healthcare use, intermediate use of healthcare and children with frequent use of healthcare. The thresholds were set in reference to the distribution of annual healthcare encounters for the analytical cohort, which had 11.0 ± 4.9 encounters per year. The classification was used for all and was not meant as a validated national benchmark for excessive health care use.

In multivariable logistic regression, high healthcare utilization (≥14 encounters per year) was considered the binary outcome and participants with low or moderate healthcare utilization (0–13 encounters per year) were used as the reference group.

### 2.8 Sociodemographic and household characteristics

Demographic and household data obtained from records included age, sex, urban or rural residence, parents’ education, household income, number of household members, primary caregiver, and insurance coverage. These variables were included because previous studies have shown them to be important factors affecting childhood nutrition, disease occurrence, and healthcare-seeking. Information on physical activity, sleep duration, environmental exposures, parental health conditions, and other lifestyle-related factors was not consistently available in the routinely collected health records. Therefore, it was not included in the multivariable analyses.

### 2.9 Key variables: operational definitions

#### 2.9.1 Nutritional status

Nutritional status was judged according to the age- and sex-standardized pediatric growth references in China through anthropometric measurements. Children were defined as undernourished, normal weight, overweight and obese by their anthropometric criteria as defined in the institutional health records.

#### 2.9.2 Dietary quality

Dietary quality was evaluated by a composite score (9 components) based on commonly recorded dietary habits. One point was given for each positive dietary habit and zero for any negative dietary habit. Reverse coding was done for adverse dietary indicators. The potential scores ranged from 0 to 9, where 0–2 were considered poor, 3–5 were considered moderate, and 6–9 were considered good dietary patterns. Detailed component-specific thresholds are provided in Supplementary Table S1.

#### 2.9.3 Childhood morbidity burden

Morbidity burden was calculated as the number of physician-diagnosed morbidity events per year in the observation period. The number of episodes/year was divided into 0–1 (low), 2–4 (moderate) and ≥5 (high) to categorize the children as having low, moderate, and high morbidity burden.

#### 2.9.4 Healthcare utilization

Healthcare utilization: The number of outpatient, emergency, inpatient, preventive, vaccination-related, specialist, and telehealth visits or consultations was counted, including all those that occurred during the course of a year. Participants were divided into groups with low, moderate, and high utilization based on the number of encounters per year, respectively, 0–7, 8–13, and ≥14. For multivariable logistic regression, high healthcare utilization was compared with the combined low- and moderate-utilization categories.

#### 2.9.5 Household socioeconomic status

Socio-economic status of the household was checked in the institutional health database based on parental educational level and household income category. Household income was classified as low, middle, or high based on the predetermined socio-economic categories employed in the institutional database. Since actual household-income information was not always available, the classification was determined by the institutional category recorded instead of a consistent national income threshold. Maternal education was divided into primary education or lower, secondary education and university education or higher. In contrast, paternal education was divided into lower than secondary education, secondary education and higher than secondary education.

### 2.10 Statistical analysis

IBM SPSS Statistics version 29.0 (Armonk, NY, United States: IBM Corp.) was used for statistical analysis. No *a priori* sample-size calculation or statistical power analysis was performed because this was a retrospective study based on routinely available health records. The study included all eligible participants identified during the predefined study period, resulting in a final analytical cohort of 720 children. Data completeness and normality were assessed using the Shapiro–Wilk test, histograms, and Q–Q plots. Data on continuous variables were displayed as mean ± standard deviation (SD) or median (interquartile range [IQR]), and categorical variables were shown as frequencies and percentages.

Normally distributed variables were tested with the independent-samples t-test or one-way Analysis of Variance (ANOVA); non-normally distributed variables were tested with the Mann–Whitney U or Kruskal–Wallis test. Categorical variables were analyzed using the Pearson chi-square test or Fisher’s exact test, depending on sample size. The Cochran-Armitage test for trend was used to assess known-groups validity of the dietary-quality score by examining whether individual dietary components varied monotonically across the derived Poor, Moderate, and Good tiers. Univariable logistic regression analyses were performed to estimate crude odds ratios (ORs) and 95% confidence intervals (CIs) for associations between candidate variables and the study outcomes.

Univariable logistic regression was conducted for each study outcome separately (high morbidity burden and high healthcare utilization). All candidate predictors were then calculated using crude odds ratios (ORs), 95% confidence intervals (CIs) and *p*-values. Variables with a *p* value < 0.20 in the respective univariable analysis were included in the respective multivariable logistic regression model. Both outcomes were analyzed using the same variable-selection procedure (12). Adjusted odds ratios (AORs) with 95% CIs were presented.

Two multivariable binary logistic regression models were constructed to identify factors independently associated with high morbidity burden and high healthcare utilization. High morbidity burden was defined as ≥5 morbidity episodes per year and was compared with low-to-moderate morbidity burden (0–4 episodes per year). High healthcare utilization was defined as ≥14 healthcare encounters per year and was compared with low-to-moderate healthcare utilization (0–13 encounters per year). Adjusted odds ratios (AORs) with 95% confidence intervals (CIs) were reported. Multicollinearity was examined using variance inflation factors (VIFs) and tolerance statistics, and model fit was evaluated using the Hosmer–Lemeshow goodness-of-fit test, Nagelkerke R^2^, classification accuracy, and area under the receiver operating characteristic curve (AUC) for the healthcare-utilization model.

Nutritional status was initially classified into four categories: undernutrition, normal nutritional status, overweight, and obesity. Normal nutritional status was taken as the reference category in the regression analyses. Univariable analyses were used to assess all nutritional status categories, but only those that survived the backward selection process during multivariable analyses are shown in the forest plots. All statistical tests were two-sided, and *p*-values < 0.05 were considered statistically significant. No formal adjustment for multiple comparisons was done, as the analyses were hypothesis-driven and variables were predetermined. Effect sizes (AORs) and 95% CIs were also used, in addition to *p*-values, to interpret associations.

### 2.11 Missing data

Records with missing information on key anthropometric, nutritional, morbidity, or healthcare-utilization variables were excluded during participant selection. Therefore, the final analytical dataset contained complete information for the primary study variables, and no statistical imputation was performed.

### 2.12 Ethical considerations

This study was approved by the Ethics Committee of Beijing Anzhen Nanchong Hospital (Approval no. 2026024). The Ethics Committee waived the need for individual informed consent, based on previously collected and anonymised data from children’s health records, in accordance with institutional and national regulations. Hence, informed consent of parents/legal guardians was not deemed necessary for secondary analysis of de-identified data.

When extracting data, all direct personal identifiers were deleted or replaced with study-specific identification codes. There was no identifiable information, such as names, addresses, contact details, etc. in the research data. Only authorized members of the research team were able to access the data, and the extracted data were kept securely and used only for this study. This study was carried out following the standards of the Declaration of Helsinki and the amendments thereto and the appropriate institutional and national guidelines for research involving human subjects and dealing with pediatric health data.

## 3 Results

### 3.1 Participant characteristics

A total of 720 children were included in the final analysis. As illustrated in Table 1, 38.3% of children were aged 6–9 years, and 38.3% were aged 10–14 years, with slightly more males than females (53.1% vs. 46.9%). The majority of participants lived in urban areas (65.6%), belonged to the middle household-income category (54.0%), and were primarily cared for by their parents (77.9%). Of the children, 64.6% were nutritionally normal, while 15.1% were undernourished, and 20.3% were overweight or obese. Moderate dietary quality was seen in almost half of the participants (47.4%), and poor dietary quality in 24.0%. The mean body mass index (BMI) was 18.2 ± 3.4 kg/m^2^. The mean height-for-age z-score was −0.11 ± 1.08, and the mean weight-for-age z-score was 0.07 ± 1.14.

**Table 1:** Baseline sociodemographic, household and nutritional characteristics of participants (*N* = 720).

Variable	Category	*n* (%)
Age Group (years)	3–5	168 (23.3)
	6–9	276 (38.3)
	10–14	276 (38.3)
Sex	Male	382 (53.1)
	Female	338 (46.9)
Residence	Urban	472 (65.6)
	Rural	248 (34.4)
Household income	Low	181 (25.1)
	Middle	389 (54.0)
	High	150 (20.8)
Family size	≤3 members	159 (22.1)
	4–5 members	404 (56.1)
	≥6 members	157 (21.8)
Primary caregiver	Parents	561 (77.9)
	Grandparents	128 (17.8)
	Others	31 (4.3)
Health insurance coverage	Yes	648 (90.0)
	No	72 (10.0)
Maternal education	Primary or less	141 (19.6)
	Secondary	329 (45.7)
	University or above	250 (34.7)
Paternal education	Primary or less	118 (16.4)
	Secondary	341 (47.4)
	University or above	261 (36.3)
Nutritional status	Undernourished	109 (15.1)
	Normal	465 (64.6)
	Overweight	89 (12.4)
	Obese	57 (7.9)
Dietary quality	Poor	173 (24.0)
	Moderate	341 (47.4)
	Good	206 (28.6)

Values are presented as *n* (%). Nutritional status was categorized according to age- and sex-specific anthropometric criteria.

### 3.2 Validity of the dietary quality score

An intra-study test of the internal consistency of the study-specific dietary-quality score compared the prevalence of each of the 9 individual dietary components across the three categories of the dietary-quality score, Poor, Moderate, and Good. All 9 components were monotonically and significantly higher in all tiers in the expected direction (Cochran-Armitage test for trend, *p* < 0.001 for all comparisons; Table 2). Favorable behaviors (daily fruit consumption, daily vegetable, dairy, regular breakfast, whole grain and adequate dietary diversity) showed progressive improvements from the Poor to the Good group, ranging from 39.3 to 67.3 percentage points. In contrast, negative behaviors (consumption of sugar sweetened beverages, fast-food, and processed-snacks) decreased across all tiers, ranging from 40.0–46.3 percentage points. An external convergent measure of the validity for the derived dietary-quality categories was also found: the more time children spent on a screen, the lower their dietary quality score was (66.5% in the Poor tier vs. 29.1% in the Good tier, *p* < 0.001), indicating a monotonic decrease across tiers.

**Table 2:** Known-groups validity of the dietary quality score: Prevalence of individual dietary components across derived tiers.

Component	Poor%	Moderate%	Good%	Gap (pp)	RR (Good/Poor)	*p*-trend
Daily fruit intake	31.8	58.9	84.0	+52.2	2.64	<0.001
Daily vegetable intake	27.2	55.4	80.6	+53.4	2.96	<0.001
Dairy ≥5 d/wk	29.5	53.1	76.2	+46.7	2.58	<0.001
Regular breakfast	52.0	76.8	91.3	+39.3	1.76	<0.001
Whole-grain consumption	18.5	41.4	68.0	+49.5	3.68	<0.001
Sugary beverages ≥3/wk	61.8	36.4	15.5	−46.3	0.25	<0.001
Fast food ≥2/wk	54.3	30.8	12.6	−41.7	0.23	<0.001
Processed snacks ≥4/wk	58.4	34.9	18.4	−40.0	0.32	<0.001
Dietary diversity ≥5 groups	22.5	63.3	89.8	+67.3	3.99	<0.001

Gap (pp) = percentage-point difference between Good and Poor tiers. RR = relative risk of the favorable/target behavior comparing Good vs. Poor tier. *p*-trend from Cochran-Armitage test for trend across ordered tiers (Poor < Moderate < Good).

### 3.3 Childhood morbidity patterns

Childhood morbidity was high in the study period, with the most prevalent diseases being respiratory and gastrointestinal tract infections. As shown in Table 3, the most commonly reported condition was upper respiratory infections, which were reported by over half of respondents (54.0%), followed by gastrointestinal disorders (29.7%) and allergic diseases (25.3%). Other common conditions reported included nutritional deficiency disease, recurrent febrile diseases and oral health diseases.

**Table 3:** Distribution of childhood morbidity patterns (*N* = 720).

Morbidity category	*n* (%)
Upper respiratory infections	389 (54.0)
Gastrointestinal disorders	214 (29.7)
Allergic diseases	182 (25.3)
Nutritional deficiency disorders	123 (17.1)
Dermatological diseases	101 (14.0)
Ear, nose and throat conditions	92 (12.8)
Asthma/wheezing disorders	87 (12.1)
Oral and dental conditions	119 (16.5)
Recurrent febrile illnesses	167 (23.2)
Injury-related healthcare visits	76 (10.6)
No documented illness episode	96 (13.3)

An assessment of cumulative disease occurrence showed that the greatest proportion of children had a moderate disease burden. Table 4 shows that 41.8% of participants had 2–4 episodes of illness per year, 25.7% had 5 or more episodes of illness per year, and were considered to be at high morbidity risk.

**Table 4:** Morbidity burden classification among participants.

Morbidity burden	Definition	*n* (%)
Low	0–1 episodes/year	234 (32.5)
Moderate	2–4 episodes/year	301 (41.8)
High	≥5 episodes/year	185 (25.7)

**Figure 2:**
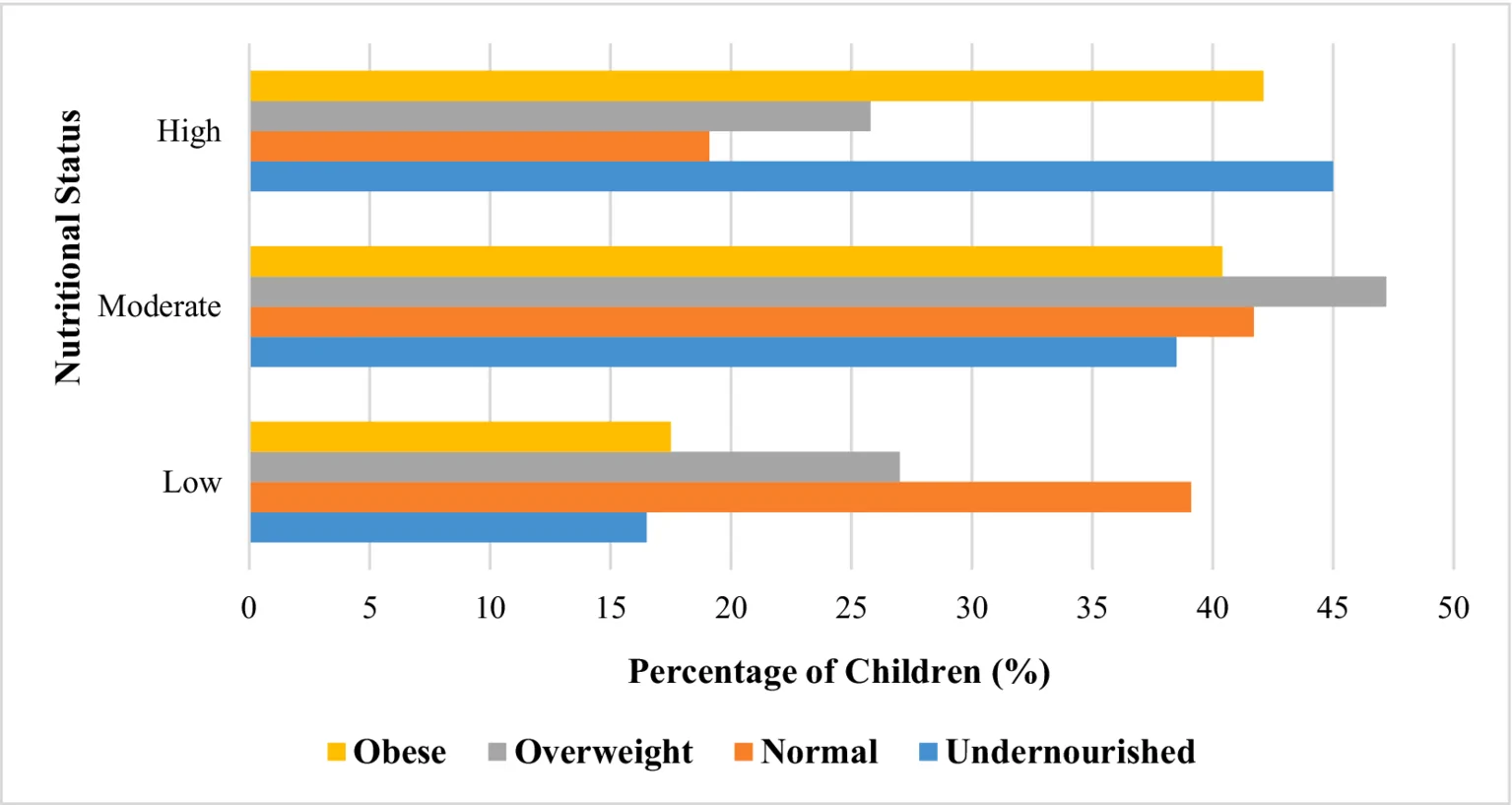
Distribution of morbidity burden according to nutritional status among Chinese children (*N* = 720).

Morbidity burden differed significantly according to nutritional status (Figure 2). A higher proportion of children with undernutrition (45.0%) and obesity (42.1%) had high morbidity burden compared with children with normal nutritional status (19.1%; *p* < 0.001).

Clustered bar chart showing the distribution of low, moderate, and high morbidity burden across nutritional status categories. Undernourished and obese children exhibited the highest proportions of high morbidity burden, whereas children with normal nutritional status were more likely to experience a low morbidity burden.

### 3.4 Nutritional status and socioeconomic characteristics

There was marked variation in nutritional status by age, sex, household income and place of residence (Table 5). The prevalence of undernutrition was higher in 3-5-year-old children, and obesity was relatively higher in 10–14-year-old children (*p* = 0.018). Undernutrition prevalence was higher among the female children whereas overweight and obesity prevalence was higher among the male children (*p* = 0.041). Children in low-income families and rural communities were also more likely to be undernutrition, while overweight and obesity were more likely in higher income families and urban communities (*p* < 0.001 and *p* = 0.002, respectively).

**Table 5:** Nutritional status according age, sex and socioeconomic characteristics.

Variable	Category	Undernourished *n* (%)	Normal *n* (%)	Overweight *n* (%)	Obese *n* (%)	*p*-value
Age group (years)	3–5	32 (19.0)	109 (64.9)	17 (10.1)	10 (6.0)	0.018
	6–9	40 (14.5)	180 (65.2)	35 (12.7)	21 (7.6)	
	10–14	37 (13.4)	176 (63.8)	37 (13.4)	26 (9.4)	
Sex	Male	53 (13.9)	244 (63.9)	52 (13.6)	33 (8.6)	0.041
	Female	56 (16.6)	221 (65.4)	37 (10.9)	24 (7.1)	
Household income	Low	52 (28.7)	101 (55.8)	19 (10.5)	9 (5.0)	<0.001
	Middle	56 (14.4)	260 (66.8)	45 (11.6)	28 (7.2)	
	High	10 (6.7)	104 (69.3)	23 (15.3)	13 (8.7)	
Residence	Rural	53 (21.4)	152 (61.3)	26 (10.5)	17 (6.8)	0.002
	Urban	56 (11.9)	313 (66.3)	63 (13.3)	40 (8.5)	

Values are presented as *n* (%). Percentages were calculated within each category. *p*-values were obtained using the Pearson chi-square test.

### 3.5 Healthcare utilization patterns

Healthcare service use was high across the study population. Outpatient services were the most commonly used health care service, averaging 4.8 visits per year (Table 6). Preventative health visits and specialist consultations were also frequently reported, as was hospital admission, but less so. The average total number of health care visits was 11.0 ± 4.9 visits per child per year.

**Table 6:** Healthcare utilization indicators among study participants.

Healthcare indicator	Mean ± SD
Outpatient visits/year	4.8 ± 2.7
Emergency visits/year	1.2 ± 1.1
Specialist consultations/year	1.8 ± 1.4
Preventive health visits/year	2.6 ± 1.3
Vaccination-related visits	1.7 ± 0.8
Telehealth consultations	0.9 ± 1.0
Hospital admissions/year	0.6 ± 0.8
Hospital length of stay (days)	3.9 ± 2.4
Total healthcare encounters	11.0 ± 4.9

Overall healthcare utilization was classified according to the annualized number of healthcare encounters. As shown in Table 7, 35.3% of participants had low healthcare utilization, defined as 0–7 encounters per year, while 38.9% had moderate utilization, defined as 8–13 encounters per year. High healthcare utilization, defined as ≥14 healthcare encounters per year, was observed in 25.8% of children.

**Table 7:** Healthcare utilization category among study participants.

Category	Annual healthcare encounters	*n* (%)
Low	0–7 encounters/year	254 (35.3)
Moderate	8–13 encounters/year	280 (38.9)
High	≥14 encounters/year	186 (25.8)

Total healthcare utilization was defined as the annual number of outpatient visits, emergency department visits, inpatient stays, preventive care visits, vaccination visits, specialist visits, and telehealth visits recorded. The categories were established by using a threshold defined by the distribution of annual healthcare encounters for each cohort. In multivariable logistic regression, high healthcare utilization (more than 14 visits per year) was compared to low and moderate utilization (0–13 visits per year).

There were considerable differences in healthcare utilization by nutritional status (Table 8). Nearly half of undernourished children (46.8%) and obese children (47.4%) had a high level of health care use, significantly more than children with normal nutritional status (18.1%) (*p* < 0.001). The results of this study suggest that undernutrition as well as obesity are linked to higher utilization of health services.

**Table 8:** Healthcare utilization according to nutritional status.

Nutritional status	Low utilization *n* (%)	Moderate utilization *n* (%)	High utilization *n* (%)	*p*-value
Undernourished	19 (17.4)	39 (35.8)	51 (46.8)	<0.001
Normal	198 (42.6)	183 (39.4)	84 (18.1)	
Overweight	25 (28.1)	40 (44.9)	24 (27.0)	
Obese	12 (21.1)	18 (31.6)	27 (47.4)	

### 3.6 Univariable analysis of factors associated with high morbidity burden

Undernutrition, obesity, poor dietary quality, rural living, low household income, larger family size, grandparent care and low maternal education level were significantly associated with high morbidity burden in univariate logistic regression analysis. Higher odds of high morbidity burden were seen with no fruit intake per day. Variables with a univariable *p*-value of <0.20, and clinically relevant variables that were garnered from the literature, were considered for the multivariable logistic regression model (Table 9).

**Table 9:** Univariable logistic regression analysis of factors associated with high morbidity burden.

Variable	Crude OR	95% CI	*p*-value
Age 6–9 years	0.86	0.54–1.37	0.526
Age 10–14 years	0.91	0.57–1.45	0.689
Male sex	1.21	0.87–1.68	0.257
Undernutrition	3.47	2.18–5.52	<0.001
Overweight	1.47	0.88–2.46	0.140
Obesity	3.08	1.70–5.58	<0.001
Poor dietary quality	3.42	2.35–4.98	<0.001
Rural residence	1.72	1.21–2.45	0.003
Low household income	2.21	1.49–3.28	<0.001
Large family size (≥6 members)	1.58	1.08–2.31	0.019
Grandparent caregiver	1.74	1.14–2.65	0.010
Daily fruit intake	0.65	0.48–0.88	0.005
Maternal university education	0.56	0.36–0.87	0.010

Age 3–5 years, female sex, normal nutritional status, good/moderate dietary quality, urban residence, middle/high household income, family size ≤5 members, parental caregiving, no daily fruit intake, and maternal primary education.

### 3.7 Factors associated with high morbidity burden

Several independent risk factors for a high level of morbidity burden were identified using multivariable logistic regression analysis. Poor dietary quality was the strongest nutritional predictor and was associated with 2.76-fold higher odds of high morbidity burden (AOR = 2.76, 95% CI: 1.89–4.05; *p* < 0.001). Other factors independently associated with increased morbidity burden included undernutrition, obesity, low household income, rural living, large family size, and grandparent caregiving. Conversely, daily fruit consumption and maternal education were protective factors associated with lower odds of high morbidity burden. The regression model demonstrated acceptable calibration (Hosmer–Lemeshow *p* = 0.512), explained 31.8% of the variation in high morbidity burden (Nagelkerke *R*^2^ = 0.318), and correctly classified 76.8% of participants.

**Figure 3:**
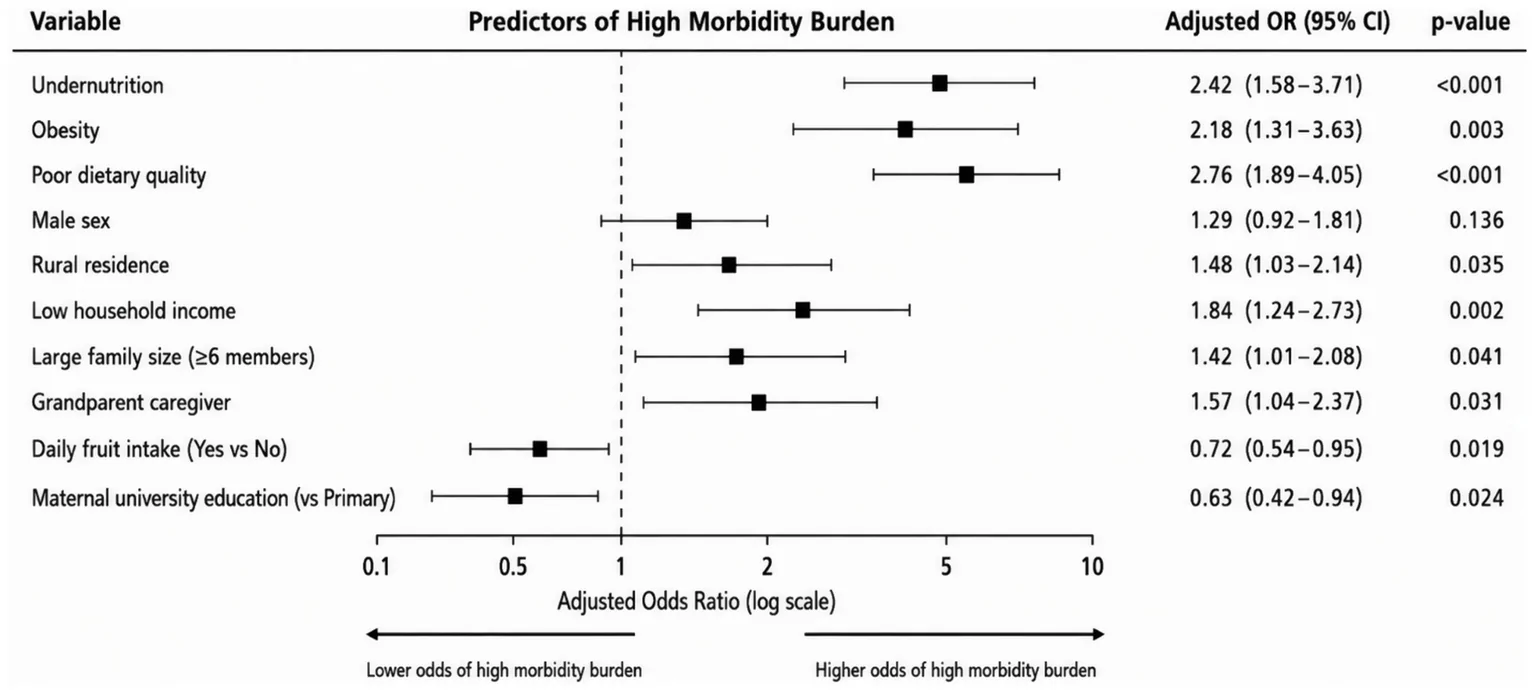
Forest plot of adjusted odds ratios for predictors of high morbidity burden.

The adjusted associations between the included predictors and high morbidity burden are presented in Figure 3, which indicates that good diet quality, undernutrition, obesity and low socio-economic factors were independently associated with higher odds of a high morbidity burden. In comparison, fruit consumption and higher maternal education were independently associated with lower odds of a high burden of morbidity.

#### 3.7.1 Reference categories

Normal nutritional status for nutritional status; good dietary quality for dietary quality; female sex for sex; urban residence for place of residence; high household income for household-income category; non-grandparent caregiving for caregiver type; ≥6 family members for family size; no daily fruit intake for daily fruit intake; and primary education for maternal education. AOR, adjusted odds ratio; CI, confidence interval.

### 3.8 Univariable analysis of factors associated with high healthcare utilization

The relationships between each of the candidate variables and high healthcare use were assessed using univariable logistic regression. The morbidity burden, grandparent care, low household income, undernutrition, obesity, rural residence, and lack of health insurance were associated with higher odds of high healthcare utilization. All variables that had a *p* < 0.20 in the univariable analysis were included in the multivariable logistic regression model for high healthcare utilization (Table 10).

**Table 10:** Univariable logistic regression analysis of factors associated with high healthcare utilization.

Variable	Crude OR	95% CI	*p*-value
Age 6–9 years	0.93	0.58–1.49	0.768
Age 10–14 years	1.08	0.68–1.72	0.747
Male sex	1.14	0.82–1.59	0.432
High morbidity burden	4.62	3.25–6.57	<0.001
Undernutrition	3.97	2.48–6.35	<0.001
Overweight	1.67	0.98–2.85	0.059
Obesity	4.09	2.24–7.46	<0.001
Poor dietary quality	2.91	1.99–4.25	<0.001
Rural residence	1.43	1.01–2.03	0.046
Low household income	1.82	1.22–2.71	0.003
Large family size (≥6 members)	1.29	0.87–1.92	0.207
Grandparent caregiver	1.68	1.08–2.60	0.021
No health insurance	1.79	1.01–3.16	0.046

Reference categories were age 3–5 years, female sex, low-to-moderate morbidity burden, normal nutritional status, good/moderate dietary quality, urban residence, middle/high household income, family size ≤5 members, parental caregiving, and health-insurance coverage. Variables with *p* < 0.20 were entered into the multivariable logistic regression model.

### 3.9 Factors associated with high healthcare utilization

Variables with *p* < 0.20 in the univariable logistic regression analysis were entered into the multivariable logistic regression model to identify factors independently associated with high healthcare utilization. High morbidity burden had the strongest association with higher rates of health service consumption, with children in the high morbidity burden group having nearly 4 times higher odds of being in the high utilization group (AOR = 3.87, 95% CI: 2.71–5.54). Undernutrition, obesity, poor dietary quality, low household income, and grandparent caregiving were also important factors predicting greater use of health services. The final model had a good discriminatory capacity (AUC = 0.812), and 79.4% of the participants were correctly classified.

**Figure 4:**
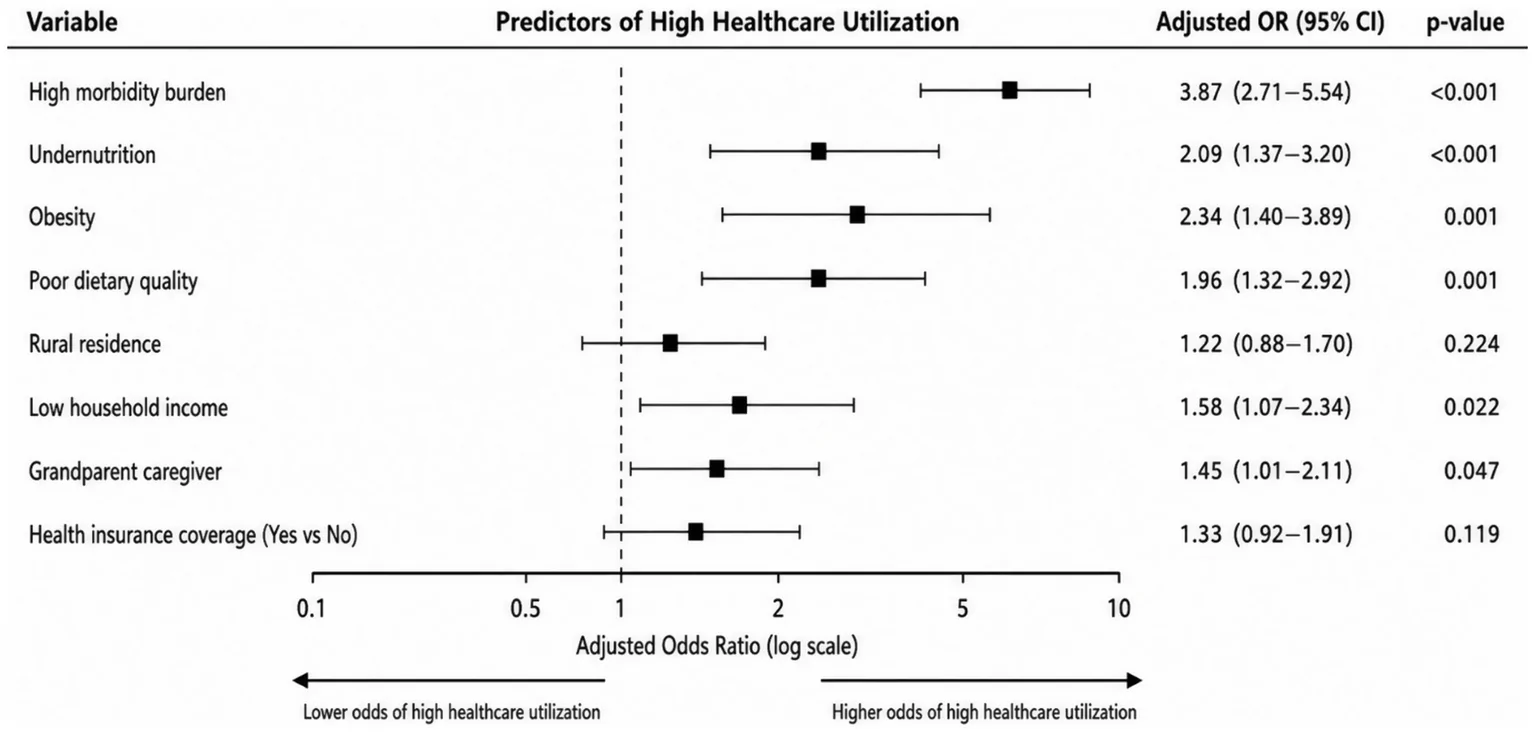
Forest plot of adjusted odds ratios for predictors of high healthcare utilization.

The healthcare-utilization model demonstrated acceptable calibration (Hosmer–Lemeshow *p* = 0.614), explained 35.6% of the variation in high healthcare utilization (Nagelkerke *R*^2^ = 0.356), achieved an area under the receiver operating characteristic curve of 0.812, and correctly classified 79.4% of participants. The adjusted associations between the included predictors and high healthcare utilization are presented in Figure 4, highlighting the very strong relationship between high morbidity burden and high levels of healthcare service use.

Low-to-moderate morbidity burden for morbidity burden; normal nutritional status for nutritional status; good dietary quality for dietary quality; urban residence for place of residence; high household income for household-income category; non-grandparent caregiving for caregiver type; and no health-insurance coverage for health-insurance status. AOR, adjusted odds ratio; CI, confidence interval.

## 4 Discussion

This single-centre, institution-based study identified significant associations between nutritional status, dietary quality, childhood morbidity burden and health care utilization among children receiving care at a tertiary healthcare centre in Sichuan, China. These findings should be interpreted in relation to the study population and healthcare setting and should not be assumed to represent the broader Chinese pediatric population. Undernutrition and overweight/obesity in children underscore the ongoing dual burden of malnutrition in Chinese children. The above results suggest the need for a public health approach to nutrition assessment in children that considers both undernutrition and overweight, rather than just undernutrition. The observed associations also indicate the possible usefulness of implementing nutritional and dietary assessments as part of the routine surveillance of children’s health, especially to identify children who might be at higher risk for illness or need more regular health care services (13).

The patterns of dietary behaviors in this study were highly differentiated across the diet quality categories, with higher intakes of fruits and vegetables, breakfast consumption, and dietary diversity within the better diet category, and higher intakes of sugary drinks, fast food, and screen time within the poor diet category. This differentiation is further supported by the known-groups validity analysis presented in the study, which confirmed that each of these behaviors varied significantly and monotonically across the derived dietary-quality tiers. The results are consistent with research showing that dietary changes in today’s society and a lack of dietary literacy are major determinants of poor dietary habits in Chinese children (14). The results are in line with previous studies showing that higher TV viewing is associated with poorer eating habits (15). The increased exposure to food marketing and behavioral displacement of regular meals have been suggested as potential pathways that could account for these associations and were not directly examined in the present study, so should therefore be interpreted as hypotheses for which there is previous evidence, but not supported by the current study data (16).

Respiratory and gastrointestinal infections were the predominant diseases, aligning with a recent study of children in China, which revealed that environmental exposures, hygiene factors, and immunity are central to disease patterns across the Country (17, 18). This high burden of allergic diseases is also consistent with the national trend of urbanization, increased exposure to pollution and lifestyle changes being associated with the increasing burden of allergic diseases (19). Importantly, a high proportion of children with multiple morbidities indicates that multiple episodes of disease do not occur, but that there is a cumulative health vulnerability.

The pattern observed indicates that undernutrition and obesity may indicate vulnerable groups of children with higher morbidity burden. From a clinical perspective, the finding suggests that children at either end of the nutrition spectrum can be monitored more closely and comprehensively assessed for nutrition. The results are aligned with the national evidence showing that persistent undernutrition exists among socio-economically disadvantaged groups while there is an increase in obesity in urbanized groups (20). While impaired immune function and chronic low-grade inflammation have been suggested as potential biological pathways in previous studies, they were not directly assessed in the present study. They should therefore be considered as biologically plausible hypotheses but not explanations based on the current data (21).

There was significant socioeconomic variation in the nutritional situation, with undernutrition more common in low-income and rural households and excess body weight more common in higher-income urban households. However, similar gradients have been observed in studies of child health in China, with structural inequities in access to food, health care, and parental education shaping nutrition risk profiles (22, 23). The results of the current study are consistent with the findings of Wu and Qi (24), who found that higher maternal education had a protective effect on feeding practices related to dietary quality. The increased burden experienced by grandparent carers is also likely due to intergenerational differences in these caregivers’ dietary control and health awareness, as observed in earlier caregiver studies (25).

There are important implications for healthcare plans relating to morbidity burden and healthcare utilization. Frequent or recurrent illness may lead to increased access to outpatient, specialist, emergency, and/or inpatient services, increasing demands on health systems. The observed links between undernutrition, obesity, poor dietary quality, and socioeconomic disadvantage with increased healthcare use suggest that nutritional and social factors could be used to identify children at higher risk of needing more intensive health monitoring. These results, however, suggest statistical correlations and not that nutrition directly leads to greater use of the health care system (26, 27).

The multivariable results showed that the nutritional, dietary, and socioeconomic factors were independently associated with the morbidity burden of childhood and healthcare utilization, while controlling for other factors in the models. The strength of these relationships indicates that diet quality or nutritional status could be important indicators of health vulnerability within child populations. However, a retrospective and observational study design precludes establishing the order of these associations and residual confounding may occur. Thus, the results presented should be viewed as associations and not as evidence of causality. The direction, mechanisms and clinical significance of these relationships need to be clarified by prospective cohort and intervention studies (28).

### 4.1 Strengths of the study

There are several strengths of this study that support the relevance of the findings in this participating health care setting. First, the study was based on a relatively large sample of 720 children, with data on nutritional status, dietary practices, morbidity, health services use, and household characteristics collected routinely. All children were identified from one tertiary healthcare center, but there were no children from rural areas and so this sample does not represent the nation. Second, the ability to combine nutritional, clinical, healthcare-utilization and socioeconomic measures allowed for the assessment of several interconnected pediatric health outcomes in a single analysis. Also, known-groups validity testing was conducted to provide empirical support for the internal structure of the study-specific dietary-quality score, even if there was no fully validated instrument, and all 9 dietary components in this study varied significantly and monotonically across derived tiers. Third, the impact of important measured sociodemographic and household factors was adjusted by multivariable logistic regression, but there was no attempt to control for unmeasured factors. Lastly, the use of routinely collected medical records for 5 years eliminated reliance on participants’ memory and provided longitudinally documented healthcare data.

### 4.2 Limitations of the study

However, this has some drawbacks. The retrospective study design does not allow confirmation of a causal relationship between nutritional determinants and health outcomes, but can only be interpreted in terms of association. In addition, dietary quality was calculated using a study-specific composite score based on common dietary behaviors that were routinely recorded, rather than a formally validated Chinese pediatric dietary quality instrument, detailed dietary recalls, or nutritional biomarkers. Known groups validity testing of the score provided by routinely collected data revealed that all 9 components increased or decreased in the predicted direction and extent between the different dietary-quality tiers (Cochran-Armitage *p* < 0.001 for all components); this validated the internal consistency of the score. The expected impact of any residual misclassification from this unvalidated instrument is generally toward the null, as the association between the classifications and the outcomes is likely to be non-differential with respect to the outcomes, so the odds ratios presented are likely to be more likely to underestimate the true odds ratios than to overestimate them. The score is to be interpreted as a good indicator of children’s relative diet and for ranking purposes only and not as an indicator of absolute nutrient intakes or for diagnosis of dietary adequacy. Thus, measurement error and dietary misclassification might have been present, and the apparent associations must be interpreted with caution. The sample was representative of its socioeconomic makeup, but it may not be sufficiently representative of regional variations across China’s provinces, which makes it somewhat less generalizable nationally.

Furthermore, residual confounding of observed relationships may have occurred because some potentially important factors affecting children’s health were not always available in routinely collected records. These included physical activity, sedentary behavior, sleep duration, environmental exposures, parent health status, parent lifestyle factors, and other behavioral determinants. There are a multitude of factors that may have affected nutrition status and/or morbidity or healthcare utilization that are not measured here and could have influenced the associations observed. Thus, conclusions should be drawn as associations and not causative.

Selection bias may also have occurred because the study included children with available institutional health records and complete information on the primary study variables; therefore, the findings may not fully represent children with limited access to healthcare or those without documented healthcare encounters. Finally, healthcare service utilization was based on the number of visits recorded, which may underestimate use beyond the formal health sector or over-the-counter self-medication.

### 4.3 Future recommendations

Future research should use multicenter, geographically diverse, and probability-based sampling frameworks to improve representativeness and determine whether the associations observed in this single-centre cohort are consistent across different regions and healthcare settings in China. Prospective cohort and intervention studies are needed to understand better the temporal and possible causal links between nutritional status, dietary patterns, childhood morbidity, and health care utilization. More complete and validated dietary assessment tools, such as food-frequency questionnaires, dietary recalls, and nutritional biomarkers, including formal internal-consistency reliability testing (e.g., Cronbach’s alpha) and criterion validation against biomarkers, may enhance the accuracy of dietary assessment. The contribution of relevant behavioral and environmental factors, such as physical activity, sedentary behavior, sleep duration, air-pollution exposure, and household lifestyle characteristics, should also be examined in future studies. National representativeness might be enhanced by province-stratified sampling. Intervention studies are needed to determine whether improvements in dietary quality or early nutritional support are associated with subsequent changes in childhood morbidity burden and healthcare utilization.

## 5 Conclusion

In this retrospective, single-centre, institution-based study, nutrition status, dietary quality, childhood morbidity burden, and healthcare utilization were associated. Poor diet quality, as measured by a study-specific composite index that demonstrated known-groups validity across its component behaviors, and socioeconomic disadvantage were also linked to worse health outcomes and higher health care use, as were undernutrition and obesity. Although these findings suggest the potential benefits of nutritional/dietary assessment as an integral part of routine child health monitoring, they must be interpreted with caution, as the study was retrospective and observational and therefore does not provide temporal or causal relationships. Furthermore, the single-center design, institution-based participant selection, and inclusion of children with available and complete healthcare records may limit the generalizability of the findings to the broader Chinese pediatric population. Future, multicenter, longitudinal studies with representative samples and with detailed measurements of factors related to nutrition, environment, and family should be carried out to establish temporal relationships and to determine if improved nutritional status or dietary quality is followed by decreased childhood morbidity and health care utilization.

## Data Availability

The raw data supporting the conclusions of this article will be made available by the authors, without undue reservation.
