# Supplementary material for: Nutritional determinants, childhood morbidity patterns, and healthcare utilization among Chinese children: a population-based study
Source: Front Public Health. 2026 Sep 8;14:1909080. doi: 10.3389/fpubh.2026.1909080 (PMC13593838; doi:10.3389/fpubh.2026.1909080)
Supplement: Supplementary file 1 [file Table_1.docx]

The dietary quality components, predefined thresholds, and scoring procedures are presented in Supplementary Table S1.

**Supplementary Table S1. Components and Scoring Criteria for the Study-Specific Composite Dietary Quality Score**

| **Dietary component** | **Favorable criterion** | **Score** |
| --- | --- | --- |
| Fruit intake | At least once daily | 1 |
| Vegetable intake | At least once daily | 1 |
| Dairy consumption | ≥5 days/week | 1 |
| Breakfast consumption | ≥5 days/week | 1 |
| Whole-grain consumption | ≥3 days/week | 1 |
| Dietary diversity | Consumption of ≥5 major food groups | 1 |
| Sugar-sweetened beverage consumption | <3 times/week | 1 |
| Fast-food consumption | <2 times/week | 1 |
| Processed-snack consumption | <4 times/week | 1 |

Each favorable dietary behavior was assigned 1 point, whereas failure to meet the predefined favorable criterion was assigned 0 points. For potentially adverse dietary behaviors, including sugar-sweetened beverage, fast-food, and processed-snack consumption, reverse scoring was applied. Consumption below the specified threshold received 1 point, whereas consumption at or above the specified threshold received 0 points. The component scores were summed to generate a total dietary-quality score ranging from 0 to 9, with higher scores indicating healthier dietary patterns. Scores of 0–2 were categorized as poor dietary quality, scores of 3–5 as moderate dietary quality, and scores of 6–9 as good dietary quality.
